# Real world data from the Heidelberg Registry of Chronic Singultus (HeReChroS)

**DOI:** 10.1186/s13023-026-04571-4

**Published:** 2026-09-07

**Authors:** Claudia Busch, Marco Richard Zugaj, Nelly Zental, Jens Keßler

**Affiliations:** 1 Medical Faculty Heidelberg, Department of Anesthesiology, Pain Medicine Section, Heidelberg University, Im Neuenheimer Feld 131, 69120 Heidelberg, Germany; 2 Medical Faculty Heidelberg, Institute of Medical Informatics, Heidelberg University, Heidelberg, Germany

**Keywords:** Chronic singultus, Germany, Registry, Quality of life

## Abstract

**Background and aims:**

Hiccups lasting more than one month (“chronic singultus”) is a rare disease that impairs quality of life. Its low prevalence hampers evidence generation. The new Heidelberg Registry of Chronic Singultus (HeReChroS) aims to gather epidemiological data, assess disease burden, identify care gaps, improve treatment, and contribute to a more precise clinical characterization of the disease. This analysis of real-world data seeks to characterize the current HeReChroS patient cohort.

**Methods:**

The routinely collected data of patients with chronic singultus (ICD 10: G25.3) from October 2022 to March 2025 were retrospectively entered into HeReChroS. Descriptive statistics were calculated on data of the initial visit, including demographics, medical history, and patient-reported outcomes (PROs) such as disease burden and well-being.

**Results:**

A total of 131 patients were included (104 male, 27 female). Common symptoms alongside hiccups included fatigue (90%), sleep disorders (80%), and concentration difficulties (70%). Comorbidity frequency was high (median 3, IQR 2, range 0–9, mode 2 comorbid organ systems), with gastrointestinal (75%), vascular (52%), and pulmonary (28%) conditions most common. Well-being was markedly reduced (mean 35.3, SD 22, range 0–90, on a 0–100 scale). Screening questionnaires suggested that 42% of patients were depressed, and 15% of patients reported suicidal thoughts.

**Conclusion:**

HeReChroS offers a large dataset of patients with chronic singultus, enabling in-depth analysis of this rare disease. Quality of life is markedly impaired, and management remains challenging. Expanding the registry across countries and languages would enhance the statistical power and generalizability. German Clinical Trials Registry (Number: DRKS00033956, 25 March 2024, URL: https://www.drks.de/DRKS00033956).

**Supplementary Information:**

The online version contains supplementary material available at https://doi.org/10.1186/s13023-026-04571-4.

## Introduction

Hiccups (singultus) is a widespread and little understood phenomenon [1–3]. They are classified as transient (< 48 h), persistent (48 h − 1 month), or chronic (> 1 month) [4]. The afferent path of the reflex arc consists of the vagus and phrenic nerves. The efferent path consists of the phrenic nerve (cervical 3–5) which innervates the diaphragm, the plexus branches to the scalene muscles (cervical 5–7), the recurrent laryngeal nerve to the glottis, and the intercostal nerves (thoracical 1–11) which innervate the intercostal muscles [2–6]. Gama-aminobutyric acid and dopamine act as central neurotransmitters for this reflex. When the hiccup reflex is triggered, there is a synchronized contraction of the inspiratory thoracic muscles and the diaphragm. This is followed within 35 milliseconds by an abrupt closure of the glottis, which produces the typical “hiccup” [2, 3, 7]. There are no conclusive findings on how this reflex is triggered, but it can be assumed that any mechanism that irritates or damages the components of the reflex arc is able to trigger a hiccup [2, 3]. Transient hiccups are well known to everyone, but chronic hiccups only affect a very small proportion of the population. The challenges for daily life with chronic singultus are multidimensional. First, there are physical impairments such as fatigue, nausea, and difficulty with daily activities [8]. But, chronic hiccups can also lead to problems such as insomnia, anorexia, exhaustion, weight loss, depression, unnecessary surgery, and even death [2, 9–11]. Financial burdens arise from the costs of medical assessments, medication, sick leave / loss of work, and other health-related expenses. At the same time, restrictions occur in the patient’s work life, which can lead to financial losses and job insecurity [8]. Chronic hiccups occur more frequently in patients over the age of 50 and with underlying malignant diseases [11, 12]. However, often no treatable etiology at all can be identified [13–16].

Chronic singultus thus represents a rare and under-researched condition with considerable impact on patients’ physical, psychological, and social functioning. Despite the substantial burden, clinical knowledge remains limited, and there is a lack of systematic data on the epidemiology, symptomatology, comorbidities, and effective treatments. As with other rare diseases, access to expertise and specialized care is limited [17], and the generation of robust evidence is challenging due to the low prevalence [17, 18]. To address these knowledge gaps and to provide a foundation for improved patient management, the Heidelberg Registry of Chronic Singultus (HeReChroS) was established by the Center for Chronic Singultus in Heidelberg, Germany.

The aim of this report is to introduce HeReChroS and to provide a descriptive characterization of the initial cohort of patients from the Singultus Center outpatient clinic in Heidelberg, focusing on demographics, comorbidities, medications, disease burden, and well-being.

## Methods

### Ethics

HeReChroS was approved by the Ethics Committee of the Medical School of Heidelberg (S-014/2024) and registered with the German Clinical Trials Registry (DRKS00033956). The registry complies with national regulations and the Declaration of Helsinki (latest revision). Informed consent was obtained from patients before adding their data to the registry, whenever possible.

### Study design

This is a retrospective, single-center, non-blinded analysis of real-world healthcare registry data. The study was conducted using data collected within the HeReChroS registry. The present report focuses exclusively on data from patients’ initial visit at the center, i.e. no follow-up or longitudinal data were included in this analysis.

### Setting

The Center for Chronic Singultus, established in 2017, is part of the Center for Rare Diseases at the University Hospital of Heidelberg (UKHD) and is the only specialized facility in Germany for diagnosing and treating chronic singultus. Patients from across the country receive care either in person or by telephone. Since 2022, all patients have completed a standardized, PROMs-based medical history form at intake, followed by a shorter, standardized form at each follow-up. In 2025, all existing data from October 2022 to March 2025 were retrospectively transferred into the newly created electronic HeReChroS. As of March 2025, all patient contacts are documented exclusively and digitally within this registry. Children and patients unable to provide consent themselves are included into the register, if that consent can be obtained from the parents/legal guardians or legally authorized representatives. If consent cannot be obtained from the patient or, where applicable, from the legal guardian/representative, the patient receives clinical care, but their data will not be entered into the analyzable registry dataset and will not be evaluated, reported, or shared for research purposes. This article analyzes only data from patients’ initial encounter with the center.

### Patient eligibility

The inclusion criteria to HeReChroS were: patients with chronic singultus (ICD 10: G25.3), contact with the Center for Chronic Singultus at the Heidelberg University Hospital. Exclusion criteria comprised refusal to provide informed consent by the patient or legal guardian, and insufficient proficiency in the German language.

### Outcome measures

All outcome measures were collected using predefined response formats. Demographic variables included age, sex, height, and weight. Age was recorded in years, sex was categorized as female, male, or diverse, height was documented in centimeters, and weight in kilograms.

Medical history was assessed using predefined dichotomous response options. Comorbidities were recorded as present or absent for each of the following categories: pulmonary, cardiac, vascular, gastrointestinal, neoplastic, cerebral, thyroid, renal, hepatic, mental health, diabetes, and cervical spine disorders. Allergies and previous surgeries were documented as free-text entries. Lifestyle-related factors were assessed as yes/no variables and included smoking, illicit drug use, vitamin supplementation, bodybuilding, and testosterone use.

Current medication was recorded as present or absent for each medication class. These included analgesics, cardiovascular medication, sedatives, hypnotics or sleep medication, pulmonary medication, corticosteroids, antibiotics, chemotherapy, dopamine, proton pump inhibitors (PPI) including pantoprazole, baclofen, gabapentin, pregabalin, and antidepressants. Previous diagnostic procedures were also assessed as yes/no variables and comprised CT, MRI, endoscopy, thyroid ultrasound, neurological assessment, ENT assessment, and laboratory testing. Previous treatment for hiccups included prior pharmacological treatment, documented as a medication list, prior non-pharmacological therapy, assessed as yes/no, as well as previous interventions and surgeries, both documented as free-text entries. A family history of hiccups was recorded dichotomously as present or absent.

Hiccup characteristics included the year of onset, hiccup frequency, breaks between hiccup episodes, and the occurrence of hiccups during sleep. Year of onset was recorded as a numerical year. Hiccup frequency was assessed using ordered categories: hiccup salvos or bursts, 0–10 hiccups per minute, 10–30 hiccups per minute, 30–60 hiccups per minute, and continuous singultus. Breaks between hiccup episodes were categorized as pauses lasting several hours up to one day, 1–3 days, 3–7 days, or 1–3 weeks. Hiccups during sleep were assessed as a yes/no variable.

Accompanying symptoms were recorded as present or absent for each symptom. These included nausea, fatigue, insomnia, meteorism, dyspnea, pain, dizziness, loss of appetite, depressed mood, sleep disturbance, social withdrawal, and concentration difficulties.

Patient-reported outcome measures included singultus severity, subjective symptom burden, and subjective well-being. Singultus severity was assessed using a numeric rating scale ranging from 0 to 10 in response to the question, “How intense or severe are the hiccup symptoms?”, with 0 indicating “not at all” and 10 indicating “strongest hiccups.” Subjective symptom burden was assessed using a numeric rating scale from 0 to 10 in response to the question, “How burdensome do you perceive your symptoms to be?”, with 0 indicating “no burden” and 10 indicating the “greatest imaginable burden.” Subjective well-being was assessed using a numeric rating scale from 0 to 100 in response to the question, “How would you rate your general well-being?”, with 0 indicating the “worst imaginable condition” and 100 indicating the “best imaginable condition.”

Psychometric screening was performed using the German short version of the Depression Anxiety Stress Scales (DASS) [19, 20]. The questionnaire comprises 21 items, with seven items assigned to each of the three subscales: depression, anxiety, and stress. Each item is rated on a 4-point scale from 0 to 3, with higher scores indicating greater symptom burden. Response options range from 0, indicating that the item did not apply at all, to 3, indicating that the item applied very much or most of the time. Problematic raw score cut-offs were defined as > 10 for depression, > 6 for anxiety, and > 10 for stress. Suicidality was additionally assessed as a dichotomous yes/no variable.

Current treatment recommendations were documented as present or absent for each recommended treatment option. Pharmacological recommendations included PPI, baclofen, gabapentin, pregabalin, antidepressants, metoclopramide, and other medications. Non-pharmacological recommendations included physiotherapy, speech therapy, complementary or naturopathic treatment, relaxation techniques, physical activity, psychotherapy, invasive procedures, and other therapy recommendations. (The complete list of variables, and response options is provided in the electronic appendix using the HeReChroS Input Template).

### Data collection and registry design

Data were collected as part of a structured clinical registry designed to systematically capture information on patients presenting with singultus. Standardized case report forms (CRFs) were used throughout. A comprehensive form was completed at the initial patient visit, while shorter, standardized follow-up forms were used for subsequent visits. This analysis focuses exclusively on data collected during the initial visit. Data collection adhered to institutional security policies and was reviewed and approved by the Information Security Officer of the UKHD. The IT infrastructure was based on an electronic data capture (EDC) proxy operating within a secure demilitarized zone, protected by three layers of firewall security and a reverse proxy system. All data entries were subject to validation, and only authenticated submissions were securely transferred into the internal EDC database.

### Data preparation, data access, and data cleaning

Data were exported from the clinical registry into a structured database for analysis (Excel; Microsoft Corp.; Seattle, WA, USA). Before statistical evaluation, the dataset was systematically prepared through consistency checks. Data cleaning included identification and removal of implausible or duplicate entries and verification of outliers against source records when applicable. Missing values were checked against the original medical records, explicitly flagged and left blank, primarily due to incomplete patient responses. To ensure data quality, all entries were independently reviewed and verified by two investigators (MZ and CB). All co-authors had full access to the dataset, and data handling followed institutional data protection standards.

### Statistical analysis

Statistical analysis was performed using SPSS (IBM Corp.; Armonk, NY, USA). No formal statistical hypotheses were predefined in the study protocol. Categorical variables were analyzed using absolute and relative frequencies, reported as percentages. For continuous variables, the analysis approach was based on distribution characteristics. Normally distributed variables are reported as mean, standard deviation (SD), and range, whereas non-normally distributed variables are reported as median, interquartile range (IQR), mode, and range.

### Manuscript preparation

This report was prepared in accordance with the Reporting of studies Conducted using Observational Routinely-collected health Data Guideline (RECORD) [21]. Artificial intelligence (DeepL; DeepL SE; Cologne, Germany) was used to translate the first draft of the manuscript into English. After that translation, the authors reviewed and edited the content, and the manuscript was proof-read by a native English-speaking researcher familiar with registry studies. The authors alone take full responsibility for the content of the publication.

## Results

Between October 2022 and March 2025, 131 patients presented for the first time to the Center for Chronic Singultus and were included in the registry. Of these, 104 (79%) identified as male, 27 (21%) as female, and none as diverse. Demographic characteristics of patients included in the register are shown in Table 1. The mean (SD, range) age was 63 (18, 20–87). The median (IQR, range) duration of symptoms prior to first contact was 19 (59, 1-650) months. Before presenting to the center, 17% of patients (14/81) had received no pharmacological treatment for hiccups, and 81% (64/79) had received no non-pharmacological treatment for hiccups.

**Table 1:** Demographic characteristics

	Mean	SD	Range	Missing
Age (years)	63	18	20–87	-
High (cm)	172.9	9.2	149–198	52
Weight (kg)	77	16	45–120	52
BMI (kg/m^2^)	25.8	4.9	17.4–42.3	52

Hiccup patterns varied both between and within individuals. The majority of patients (85/122 (70%)) reported hiccup-free intervals lasting between one hour and one day. A minority of patients (28/122 (23%)) reported symptom-free periods lasting several days, while another small subset (9/122 (7%)) experienced continuous hiccups without any pause. Hiccup frequency ranged widely, from intermittent bursts to rates of 10, 30, or even 60 hiccups per minute. Subjective hiccup intensity rating (on a 0–10 NRS) was high with a mean (SD, range) score of 8.2 (1.8, 0–10). The subjective disease burden (on a 0–10 NRS) was also high with a mean (SD, range) of 8.3 (2.0, 0–10). Hiccups during sleep were reported by 80/125 patients (64%). A family history of hiccups was reported by 6/118 (5%) of patients, who indicated that at least one relative was also affected.

Patients showed a high burden of comorbidities (Tables 2 and 3). Median (IQR, range, mode) comorbid organ systems was 3 (2, 0–9, 2). Patients frequently reported additional symptoms beyond hiccups (Tables 4 and 5). Median (IQR, range, mode) of reported additional symptoms were 6 (4, 0–12, 5). Previous diagnostics are summarized in Tables 6 and 7. Median (IQR, range, mode) of previous diagnostics was 4 (4, 0–8, 5). Current medication use is summarized in Tables 8 and 9. Median (IQR, range, mode) of current medication classes was 2 (2, 0–7, 1). Alcohol consumption was reported by 43/130 (33%) of patients. Regular testosterone use for muscle training was noted in 9/107 (8%), and 4/130 (3%) reported regular use of illicit drugs.

**Table 2:** List of comorbidities

Comorbidity	(%)	*n*	Missing
Gastrointestinal	74.8	98/131	
Vascular	51.9	68/131	
Pulmonary	27.5	36/131	
Diabetes	27.4	26/95	36
Psychiatric	26.2	28/107	24
Cardiac	21.5	28/130	1
Cerebral	20.6	27/131	
Thyroidal	17.8	23/129	2
Splenic	17.8	23/129	2
Cervical Spine	14.0	7/50	81
Neoplastic	12.2	16/131	
Hepatic	11.5	15/130	1

**Table 3:** Number of comorbid organ systems per patient with median (blue arrow)

Comorbid organ systems	n	(%)
0	7/131	5.3
1	23/131	17.6
2	32/131	24.4
3	20/131	15.3
4	22/131	16.8
5	12/131	9.2
6	7/131	5.3
7	7/131	5.3
8	0/131	
9	7/131	0.8

**Table 4:** List of accompanying symptoms

Accompanying Symptom	(%)	*n*	Missing
Fatigue	90.2	111/123	8
Sleep disorder	80.0	100/125	6
Concentration disorder	69.6	87/125	6
Nausea	62.6	77/123	8
Dyspnea	59.2	74/125	6
Meteorism	47.9	25/48	83
Vomiting	45.6	57/125	6
Depressive mood	45.2	56/124	7
Pain	36.6	45/123	8
Social withdrawal	33.9	42/124	7
Loss of appetite	31.2	39/125	6
Dizziness	15.2	19/125	6

**Table 5:** Number of accompanying symptoms per patient with median (blue arrow)

Accompanying symptoms	n	(%)
0	2/131	1.5
1	3/131	2.3
2	5/131	4.0
3	8/131	6.1
4	19/131	14.5
5	23/131	17.6
6	19/131	14.5
7	12/131	9.2
8	16/131	12.2
9	8/131	6.1
10	6/131	4.6
11	2/131	1.5
12	2/131	1.5
Missing	6/131	4.6

**Table 6:** List of previous diagnostics

Previous diagnostics	(%)	*n*	Missing
Oesophagus gastroscopy (EGD)	81,7	107/131	
Blood lab values	80,2	105/130	1
CT chest	53,4	70/131	
MRI cerebrum	52,7	96/131	
Sonography thyroid gland	51,1	67/131	
Neurological assessment	39,2	51/130	1
MRI neck	16,8	22/131	
ENT examination	12,2	16/131	

**Table 7:** Number of previous diagnostics per patient with median (blue arrow)

Previous diagnostics	n	(%)
0	3/131	2.3
1	11/131	8.4
2	19/131	14.5
3	16/131	12.2
4	25/131	19.1
5	22/131	16.8
6	22/131	16.8
7	9/131	6.9
8	4/131	3.1

**Table 8:** List of current medication at first contact

Current medication	(%)	*n*	Missing
PPI (Proton pump inhibitors)	54.6	71/130	1
Cardiovascular	53.1	69/130	1
Baclofen	16.2	21/130	1
Antidepressants	13.8	18/130	1
Pulmonary	11.6	15/129	2
Analgesics	11.5	15/130	1
Cortisone	11.5	15/130	1
Sleep medication	8.5	11/130	1
Sedatives	6.9	9/130	1
Dopamine agonists	6.9	9/130	1
Gabapentin	6.2	8/130	1
Pregabalin	3.9	5/129	2
Chemotherapeutics	3.8	5/130	1
Antibiotics	0.8	1/129	2

**Table 9:** Number of current medications per patient with median (blue arrow)

Current medication	n	(%)
0	27/131	20.6
1	29/131	22.1
2	28/131	21.4
3	22/131	16.8
4	12/131	9.2
5	7/131	5.3
6	4/131	3.1
7	2/131	1.5

Psychometric screening (DASS-21) showed a high mean (SD, range) for stress 9.5 (6, 0–21), for depression 9.1 (6.1, 0–21), and for anxiety 6.7 (4.8, 0–21). Elevated stress scores (S > 10) occurred in 47/116 patients (41%), elevated depression scores (D > 10) in 49/116 patients (42%), and elevated anxiety scores (A > 6) in 54/116 patients (47%). When asked to rate their general well-being on a 0–100 scale, patients reported a low mean (median, SD, range) 35.3 (30, 22, 0–90). Notably, 20/127 patients (15%) indicated having had thoughts of ending their lives due to the burden of their condition.

## Discussion

HeReChroS provides a large dataset of patients with chronic singultus, allowing detailed investigation of this rare condition. Quality of life is substantially affected, and clinical management remains challenging. The most frequent additional symptoms in our collective were: sleep disturbance (80%), fatique (90%), and difficulty concentrating (70%). These findings are consistent with previous hiccup case reports and narrative reviews [22, 23].

Soudjian, involved a “second-hit” theory, in which two or more dysfunctions along the afferent, central, or efferent arc of the hiccup reflex may contribute to disease development [22]. This theory aligns with our observation of a high comorbidity burden in affected patients (median 3 comorbid organ systems). For instance, 75% had gastrointestinal comorbidities potentially affecting the afferent limb of the reflex arc, 52% had vascular comorbidities that may compromise cerebral blood flow, including the medulla oblongata, which plays a key role in hiccup regulation, 28% had pulmonary comorbidities that may stimulate the efferent limb via the phrenic nerve, and 27% had diabetes mellitus, which is known to induce autonomic dysfunction, here potentially affecting the hiccup reflex arc. Chronic Singultus is a multidisciplinary disease involving gastroenterology, neurology, pulmology, psychology, and others. Most patients were in therapy with a gastroenterologist. That may have had an influence on pre-medication (PPI: 55%) and preexisting diagnostics (esophageogastroduodenoscopy (EGD): 82%). Cymet et al. [12] also reported gastroenterology as the most frequent discipline in threating (persistent) singultus and stated that EGDs had no impact on speeding up recovery and did not provide any additional clues for the diagnosis. However, our clinical impression is that a comprehensive diagnostic work-up should be performed for all patients, in order to rule out life-threatening causes of new onset chronic singultus. That includes at a minimum: a CT scan of the chest, endoscopy of the upper gastrointestinal tract, an MRI of the skull and neck, and ultrasounds of the abdomen and thyroid.

Only 64% of our patients reported hiccups during sleep. Some clinicians state that hiccups that stop at night have a psychological etiology; whereas, hiccups that continue at night have a somatic etiology. Our clinical impression is different: after symptomatic therapy, hiccups often stop first during the night and then later decrease during day. Importantly, fatigue, sleep disturbance, and concentration problems are also core symptoms of depression. However, a majority of 80% reported sleep disturbance and qualitative data from a prior study suggest that the remaining patients may be sleepless because of anxiety about the next hiccup attack or as symptom of depression [24].

Our data shows quantification of general well-being via NRS (0-100) with low measures (median 30 from 100). A comparison with other chronic diseases highlights the severe disease burden in our patients with chronic hiccups. The Kansas City Cardiomyopathy Questionnaire overall score, composed of five subcategories, reflects quality of life across heart failure stages. Patients with NYHA class I heart failure had a median score above 80/100, whereas those with NYHA class IV scored a median around 25/100 [25]—similar to patients with chronic hiccups.

From a prior study we know, that in addition to the accompanying symptoms such as gastroenterological complaints, fatigue, and insomnia, it is primarily emotional aspects that worsen quality of life [3]. Potential mechanisms of action also lay in social withdrawal and marginalization by health care stakeholders and peers [3]. Overall, chronic hiccups can lead to depression, anxiety, and exhaustion [9]. Our data shows for the first time a representative quantification of that phenomena: around 40% of patients with chronic hiccups have anxiety, depression, and high stress levels according to the DASS-21 screening questionnaire. For context: post-stroke patients treated in a German rehabilitation clinic had mean scores of D = 4.5, A = 3.3, and S = 6.0 (9.1, 6.7, and 9.5 in chronic singultus) [26].

This registry-based study has several inherent limitations that must be acknowledged. First, the data used were not originally collected to address the specific research questions posed in this analysis, which may limit the precision and contextual relevance of key variables. Additionally, the registry lacks information on several important confounding variables—such as medication adherence or social determinants of health—which may have influenced both the presentation and management of singultus but could not be accounted for in the analysis. Missing data further restricts the accuracy of certain outcome measures, and the missing data may not be missing randomly. Finally, changes in eligibility criteria or clinical documentation standards over time could have introduced temporal inconsistencies in case inclusion and variable completeness. The 0-100 scale of well-being is neither validated nor a Quality of Life (QoL) instrument. Further, it is difficult to interpret because there are no population norms to compare it to.

In conclusion, this new registry of chronic singultus patients should enable us to track the long-term course of illness, including symptom duration, treatment effectiveness, and interaction with comorbidities, especially psychological ones. In future, treatment goals relevant to patients’ quality of life can be identified, and sex-related differences can be examined. This will also provide the basis for a systematic evaluation of therapeutic measures over time. We are eager to expand our registry to other countries and languages, in order to have larger and more varied data available for analysis. We encourage other chronic singultus centers to contact us for further research collaboration.

## Supplementary Information

Below is the link to the electronic supplementary material.

Supplementary Material 1

## Data Availability

The anonymized data that support the findings of this study are available from the corresponding author upon reasonable request.

## Abbreviations

CRF: Standardized case form
DASS: Depression anxiety stress scale
EDC: Electronic data capture
HeReChroS: Heidelberg Registry of Chronic Singultus
NRS: Numeric rating scale
PROs: Patient reported outcomes
PROMs: Patient reported outcome measures
PPI: Proton pump inhibitor
UKHD: Universitätsklinikum Heidelberg
