# Supplementary material for: Real world data from the Heidelberg Registry of Chronic Singultus (HeReChroS)
Source: Orphanet J Rare Dis. 2026 Sep 7;21:332. doi: 10.1186/s13023-026-04571-4 (PMC13613689; doi:10.1186/s13023-026-04571-4)
Supplement: Supplementary file 1 [file 13023_2026_4571_MOESM1_ESM.docx]

# Heidelberg Registry of Chronic Singultus (HeReChroS) Input Template

| **Domain** | **Variable** | **Response options / format** |
| --- | --- | --- |
| Demographics | Age | Numeric value in years |
|  | Sex | Female; male; diverse |
|  | Height | Numeric value in centimeters |
|  | Weight | Numeric value in kilograms |
| Medical history: comorbidities | Pulmonary comorbidity | Yes / no |
|  | Cardiac comorbidity | Yes / no |
|  | Vascular comorbidity | Yes / no |
|  | Gastrointestinal comorbidity | Yes / no |
|  | Neoplasia | Yes / no |
|  | Cerebral comorbidity | Yes / no |
|  | Thyroid comorbidity | Yes / no |
|  | Renal comorbidity | Yes / no |
|  | Hepatic comorbidity | Yes / no |
|  | Mental health comorbidity | Yes / no |
|  | Diabetes | Yes / no |
|  | Cervical spine comorbidity | Yes / no |
| Medical history | Allergies | Free-text entry |
|  | Previous surgeries | Free-text entry |
| Lifestyle factors | Smoking | Yes / no |
|  | Illicit drug use | Yes / no |
|  | Vitamin supplementation | Yes / no |
|  | Bodybuilding | Yes / no |
|  | Testosterone use | Yes / no |
| Current medication | Analgesics | Yes / no |
|  | Cardiovascular medication | Yes / no |
|  | Sedatives | Yes / no |
|  | Hypnotics / sleep medication | Yes / no |
|  | Pulmonary medication | Yes / no |
|  | Corticosteroids | Yes / no |
|  | Antibiotics | Yes / no |
|  | Chemotherapy | Yes / no |
|  | Dopamine | Yes / no |
|  | Pantoprazole / proton pump inhibitors (PPI) | Yes / no |
|  | Baclofen | Yes / no |
|  | Gabapentin | Yes / no |
|  | Pregabalin | Yes / no |
|  | Antidepressants | Yes / no |
| Prior diagnostic work-up | Computed tomography (CT) | Yes / no |
|  | Magnetic resonance imaging (MRI) | Yes / no |
|  | Endoscopy | Yes / no |
|  | Thyroid ultrasound | Yes / no |
|  | Neurological assessment | Yes / no |
|  | ENT assessment | Yes / no |
|  | Laboratory testing | Yes / no |
| Previous treatment for hiccups | Prior pharmacological treatment | Medication list / free-text entry |
|  | Prior non-pharmacological therapy | Yes / no |
|  | Prior interventions | Free-text entry |
|  | Prior surgeries for hiccups | Free-text entry |
| Family history | Family history of hiccups | Yes / no |
| Hiccup characteristics | Year of onset | Calendar year |
|  | Hiccup frequency | Hiccup salvos / bursts; 0-10 hiccups/min; 10-30 hiccups/min; 30-60 hiccups/min; continuous singultus |
|  | Breaks between hiccup episodes | Pauses lasting several hours up to one day; 1-3 days; 3-7 days; 1-3 weeks |
|  | Hiccups during sleep | Yes / no |
| Accompanying symptoms | Nausea | Yes / no |
|  | Fatigue | Yes / no |
|  | Insomnia | Yes / no |
|  | Meteorism | Yes / no |
|  | Dyspnea | Yes / no |
|  | Pain | Yes / no |
|  | Dizziness | Yes / no |
|  | Loss of appetite | Yes / no |
|  | Depressed mood | Yes / no |
|  | Sleep disturbance | Yes / no |
|  | Social withdrawal | Yes / no |
|  | Concentration difficulties | Yes / no |
| Patient-reported outcome measures (PROMs) | Singultus severity | Numeric Rating Scale: "How intense or severe are the hiccup symptoms?" 0 = not at all; 10 = strongest hiccups |
|  | Subjective symptom burden | Numeric Rating Scale: "How burdensome do you perceive your symptoms to be?" 0 = no burden; 10 = greatest imaginable burden |
|  | Subjective well-being | Numeric Rating Scale: "How would you rate your general well-being?" 0 = worst imaginable condition; 100 = best imaginable condition |
| Psychometric screening | DASS-21 Depression subscale | German DASS short version; 7 items rated 0-3; raw score 0-21; problematic raw cut-off: >10 |
|  | DASS-21 Anxiety subscale | German DASS short version; 7 items rated 0-3; raw score 0-21; problematic raw cut-off: >6 |
|  | DASS-21 Stress subscale | German DASS short version; 7 items rated 0-3; raw score 0-21; problematic raw cut-off: >10 |
|  | Suicidality | Yes / no |
| Current treatment recommendations | Pantoprazole / proton pump inhibitors (PPI) | Yes / no |
|  | Baclofen | Yes / no |
|  | Gabapentin | Yes / no |
|  | Pregabalin | Yes / no |
|  | Antidepressant | Yes / no |
|  | Metoclopramide | Yes / no |
|  | Other medications | Yes / no |
|  | Physiotherapy | Yes / no |
|  | Speech therapy | Yes / no |
|  | Complementary / naturopathic treatment | Yes / no |
|  | Relaxation | Yes / no |
|  | Physical activity | Yes / no |
|  | Psychotherapy | Yes / no |
|  | Invasive procedure | Yes / no |
|  | Other therapy recommendation | Yes / no |

*Note. DASS = Depression Anxiety Stress Scales; PPI = proton pump inhibitor; ENT = ear, nose and throat. DASS item ratings: 0 = did not apply at all; 1 = applied to some degree or some of the time; 2 = applied to a considerable degree or a good part of the time; 3 = applied very much or most of the time. Treatment recommendation variables correspond to the HeReChroS input template / electronic appendix.*
